# Association Between Uric Acid Levels and Obstructive Sleep Apnea Syndrome in a Large Epidemiological Sample

**DOI:** 10.1371/journal.pone.0066891

**Published:** 2013-06-24

**Authors:** Camila Hirotsu, Sergio Tufik, Camila Guindalini, Diego R. Mazzotti, Lia R. Bittencourt, Monica L. Andersen

**Affiliations:** Departamento de Psicobiologia, Universidade Federal de Sao Paulo, Sao Paulo, Brazil; Texas A & M, Division of Cardiology, United States of America

## Abstract

**Introduction:**

Recurrent hypoxia, which is associated with obstructive sleep apnea syndrome (OSAS), leads to an increase in the degradation of adenosine triphosphatase into xanthine, which in turn increases uric acid concentrations.

**Objective:**

The current study aimed to determine whether an association exists between OSAS and uric acid levels in the peripheral blood from a representative population of Sao Paulo (Brazil).

**Methods:**

A population-based survey adopting a probabilistic 3-stage cluster sample of Sao Paulo was used to represent the population according to gender, age, and socioeconomic class. A total of 1,042 volunteers underwent polysomnography recordings for OSAS diagnosis, blood pressure assessment, and biochemical blood analysis, and answered questionnaires.

**Results:**

Uric acid levels were correlated with most important risk factors for OSAS, such as AHI, desaturation time and index, minimum oxyhemoglobin saturation (SpO_2_), blood pressure, cholesterol, BMI, triglycerides and arousal, and with OSAS itself. Also, uric acid was increased in OSAS volunteers even after controlling for all confounders. Hyperuricemic volunteers presented lower mean and minimum SpO_2_ and increased desaturation index. Importantly, minimum SpO_2_ was a significant predictor of uric acid levels, which in turn was considered an independent predictor for OSAS in the binary logistic model. However, a ROC curve analysis for establishing cut-off points for uric acid levels as a biomarker of OSAS revealed moderate sensitivity and specificity.

**Conclusion:**

A strong association was found between uric acid levels and OSAS in a representative sample of the population of Sao Paulo. Although they do not qualify for a biomarker alone, uric acid levels may be involved in OSAS severity and should be considered in sleep apnea management in the future.

## Introduction

Obstructive sleep apnea syndrome (OSAS) is a highly prevalent disorder affecting 2 to 33% of the population [1], [2] (depending on study methodology) and is associated with sympathetic activation, metabolic dysregulation, and neurocognitive changes [3], [4]. OSAS is characterized by recurrent apneas associated with cyclic changes in oxyhemoglobin saturation (SpO_2_) and alterations in heart rate as well as in blood pressure during sleep [5], [6], [7]. Epidemiologic evidence has confirmed that OSAS drastically promotes cardiovascular risks independent of age, sex, race, and other common risk factors for cardiovascular diseases such as smoking, drinking, diabetes mellitus, obesity, dyslipidemia, and hypertension (for review, see [8]). Indeed, patients with severe OSAS display a higher prevalence of coronary artery disease, heart failure, and stroke (for review, see [9]).

It is well known that the repeated upper airway obstruction episodes during OSAS produce an intermittent state of hypercapnia and hypoxia, which is accompanied by decreased blood oxygen saturation and arousals during sleep [10]. Of note, these multiple cycles of hypoxia/reoxygenation are associated with increased production of reactive oxygen species (ROS), and can alter the integrity of cellular metabolic processes [11]. Inadequate oxygen supplies can impair the formation of adenosine triphosphate (ATP), an important compound for cellular homeostasis. In response, this leads to a net degradation of ATP to adenosine diphosphate and adenosine monophosphate [12]. Thus, this process causes the release of purine intermediates (adenosine, inosine, hypoxanthine and xanthine), ending with an overproduction of uric acid, the purine final catabolic product. As a consequence, high levels of ATP degradation products have been suggested as potential markers of tissue hypoxia in neonates with infant respiratory distress syndrome [13], [14], ill patients [15], [16], exercising individuals [17], [18] and pulmonary hypertension patients [19].

Hyperuricemia has also been associated with heart failure, multiple proaterogenic processes, and hypertension [20], [21], and is considered an independent predictor of death in patients at high risk of cardiovascular disease [22]. In addition, Sahebjami [11] has shown that uric acid excretion is increased in OSAS patients and normalized after continuous positive airway pressure (CPAP) treatment, most likely reflecting an association between hyperuricemia and OSAS. Additional studies have also contributed to the hypothesis that uric acid levels and sleep-disordered breathing are related, although none have evaluated these parameters in a large and representative population [23], [24], [25]. Uric acid formation is a result of the activity of xanthine oxidase, an enzyme that plays a mechanistic role in oxidative stress and cardiovascular diseases. Its production is accompanied by the enhanced synthesis of ROS, which play a significant role in hypoxia-related tissue damage [26].

Considering that the responses to the nocturnal hypoxemia accompanying OSAS may vary among different populations, the aim of this study was to elucidate the possible association between uric acid levels and OSAS through hypoxia-related parameters such as apnea-hypopnea index (AHI), SpO_2_ and desaturation index during sleep in an epidemiological sample of Sao Paulo. Moreover, we aimed to understand the possible role of uric acid as a biomarker of OSAS, since inexpensive methods of excluding significant sleep-disordered breathing such as sleep apnea are desirable.

## Materials and Methods

### Ethics Statement

The study was approved by the local ethical committee (CEP 0593/06) and registered with ClinicalTrials.gov (Identifier NCT00596713), following the principles of the Declaration of Helsinki [27].

### The Population Investigated

The Epidemiologic Sleep Study (EPISONO) is a large epidemiological study examining sleep disturbances and their risk factors. The investigation was performed in the city of São Paulo, Brazil. When the EPISONO was conducted (2007), São Paulo had more than 10 million inhabitants. Complete rational design, sampling, and procedures have been described previously [28]. Briefly, this single center study included a total of 1,101 volunteers (men and women) from São Paulo. The selection of subjects was performed according to the 3-stage cluster sampling technique to obtain a representative sample from São Paulo as previously described [28].

At the time of selection, the volunteers signed the informed consent form and answered the home questionnaires. Of these, 1,042 agreed to undergo polysomnography (PSG); a small amount of volunteers (5.4%) refused the PSG test. Age, gender, and socio-economic status distributions did not significantly differ between the volunteers who accepted PSG recording and those who refused. The subjects arrived 2 hours before bedtime at the Sleep Institute and answered institutional questionnaires. Volunteers underwent a complete overnight PSG and blood samples were collected for biochemical analysis the following day. Those who were receiving uric acid-lowering medications (such as allopurinol or probenecid), which may impact uric acid levels, were excluded from the present analysis, totaling 1,021 volunteers.

### Physical Measurements

General physical measurements were taken immediately before the subjects were prepared for the PSG hook-up, following recommended procedures and utilizing precise instruments. Measurements were taken by 2 trained physical education teachers and included systolic blood pressure (SBP, mmHg), diastolic blood pressure (DBP, mmHg), body weight (kg), height (m) and calculation of body mass index (BMI) using the formula (weight/height^2^).

### Polysomnography

A full-night PSG was performed using a digital system (EMBLA® S7000, Embla Systems, Inc., Broomfield, CO., USA) at the sleep laboratory during the subject’s habitual sleep time according to previously described [1]. The following physiological variables were monitored simultaneously and continuously: 4 channels for the electroencephalogram (EEG); 2 channels for the electrooculogram; 4 channels for the surface electromyogram (submentonian region, anterior tibialis muscle, masseter region, and seventh intercostal space); 1 channel for an electrocardiogram; airflow detection via 2 channels through a thermocouple (one channel) and nasal pressure (one channel); respiratory effort of the thorax (one channel) and of the abdomen (one channel) using inductance plethysmography; snoring (one channel) and body position (one channel); oxyhemoglobin saturation (SpO_2_, percentage of available hemoglobin that is saturated with oxygen estimated by pulse oximetry); and pulse rate. Desaturation time was calculated through the cumulative percentage sleep time with SpO_2_≤90%. Oxygen desaturation index was calculated by dividing the total number of oxygen desaturations by the total sleep time, with desaturation defined as a ≥10s reduction in SpO_2_ (≥4% of baseline at the nadir), independent of airflow or thoracoabdominal movement.

Four trained technicians visually scored all PSGs according to standardized criteria for investigating sleep [29]. EEG arousals and leg movements were scored according to the criteria established by the AASM Manual for Scoring Sleep and Associated Events [30]. Apneas were scored and classified following the recommended respiratory rules for adults suggested by the AASM Manual, and hypopneas were scored according to the alternative rules [30]. A registered PSG technologist randomly selected and rescored the sleep stages of 4% of the PSGs in order to verify their accuracy (agreement rate of 93.3±5.1%, κ = 0.91±0.03).

### Clinical Assessment

OSAS was diagnosed according to the criteria of the International Classification of Sleep Disorders (ICSD-2) proposed by American Academy of Sleep Medicine (AASM) [31]. Subjects were diagnosed with OSAS if they had 5≤AHI≤14.9 and presented at least one of the following complaints: loud snoring, daytime sleepiness, fatigue, and breathing interruptions during sleep. Subjects with an AHI≥15 were diagnosed with OSAS regardless of whether they had any additional complaints. Loud snoring was assessed using the second question of the Berlin Questionnaire for sleep apnea [32]: a positive response included snoring that was “louder than talking” or “very loud – can be heard in adjacent rooms.” Daytime sleepiness was assessed using the Epworth Sleepiness Scale [33] and the 8 questions of the Pittsburgh Sleep Quality Index [34]; Epworth scores higher than 9 and/or frequencies greater than once a week according to the Pittsburgh Index were considered to be positive. Fatigue was assessed with the Chalder Fatigue Scale [35], and scores higher than 4 were considered to be positive. Breathing interruptions were assessed using the fifth question of the Berlin Questionnaire [32] and were considered to be positive when the frequency was “higher than once a month.”

### Ethnicity and Social Class

Individuals self-reported their ethnic origin according to the following classifications used by the Brazilian Institute of Geography and Statistics (IBGE): Caucasian, Afro-Brazilian, Mixed Race (mulattos), Asian, Native (indigenous), and unknown/other. Social class was defined as high, middle, or low according to the Brazilian Economic Classification Criteria (www.abep.org), as household incomes greater than $15,961 (US dollars), between $4561 and $15,960, and lower than $4560 per year, respectively.

### Sample Collection and Biochemical Analysis

Approximately 45 mL of venous blood was collected from subjects’ forearms after 12 hours of overnight fasting. After collection, the tubes were centrifuged at 3,500 rpm for 10 minutes. Serum was kept at room temperature and plasma was stored at 4°C. The blood analysis included the following blood exams: uric acid, creatinine, glucose, total cholesterol and its fractions (LDL and HDL), triglyceride, sodium (Na^+^) and potassium (K^+^) levels. All were obtained through automated dosages with an Advia® 1650 chemistry system (Siemens Healthcare Diagnostics Inc., USA).

### Statistical Analysis

All data that did not met the assumptions of normality and homogeneity were Z-score transformed for suitable parametric evaluation. Analyses of covariance (ANCOVA) were used to investigate the effects of OSAS on uric acid levels with control for the confounding variables: BMI, age, gender, social class, ethnicity, and cardiovascular risk factors (SBP, DBP, cholesterol and fractions, triglycerides and glucose levels). To evaluate the relationship between uric acid levels and OSAS-related risk factors, a Pearson correlation test was performed. To determine the possible predictors of uric acid levels in men, women and the total population, all the variables which presented a significant correlation with uric acid were included in a multiple linear regression model using the stepwise procedure. A Chi-square test was performed to determine the association between uricemia (normouricemic or hyperuricemic) and OSAS. Hyperuricemia was defined by the concentration ≥95th percentile of the normal distribution curve by gender. Then, binary logistic regression was used to reveal the predictors of OSAS using the backward-conditional method and Hosmer-Lemeshow goodness-of-fit. The categorical variables chosen for the logistic regression were: gender, age (≤43 or >43 years), and BMI (≤26.8 or >26.8 kg/m^2^). The continuous variables chosen for the logistic regression were: SBP, uric acid, total cholesterol, LDL, HDL, triglycerides and glucose. In addition, a receiver-operating characteristics (ROC) curve was used to determine the optimal cut-off point for uric acid as a classifier of OSAS (according to the highest sensitivity and specificity). Significance level was set at 5%. Data are reported as means ± standard error of the mean (SEM). All analyses were performed using SPSS 17 (SPSS Inc., Chicago, IL, USA).

## Results

Of the 1,042 volunteers enrolled in the study, 21 were excluded for taking medication that could affect uric acid levels. Thus, 1,021 were included in the current study. From these, 177 were missing desaturation index; 29 were missing SBP and DBP; 24 were missing desaturation time; 14 were missing triglycerides and LDL; 8 were missing Na^+^ and K^+^ levels; and 1 was missing glucose and BMI data. Of the 1,021 participants, 456 (44.7%) were men and 565 (55.3%) were women. Mean ± SEM age was 42.5±0.5 years and mean BMI was 26.8±0.2 kg/m^2^. Of the 1021 volunteers, 321 were hypertensive (defined as systolic blood pressure equal to and greater than 140 mmHg and/or diastolic blood pressure equal to and greater than 90 mmHg) and 68 reported using an antihypertensive. However, there was no significant effect on uric acid levels between hypertensive and non-hypertensive as well as antihypertensive users and non-users.

**Figure 1:**
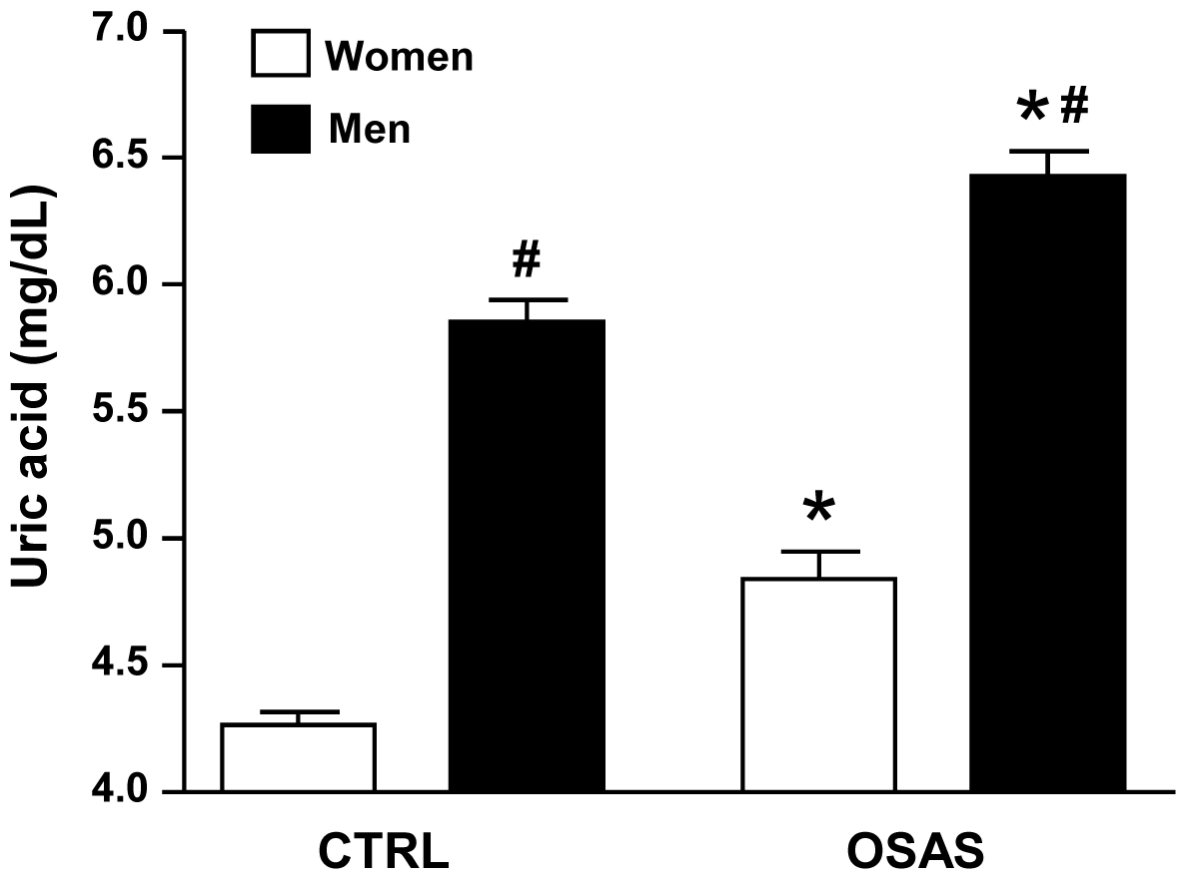
Serum uric acid levels in control (CTRL) and obstructive sleep apnea syndrome (OSAS) groups stratified by gender. Data are shown as mean±SEM. *p<0.0001 compared to respective CTRL group; #p<0.0001 compared to women gender.

**Figure 2:**
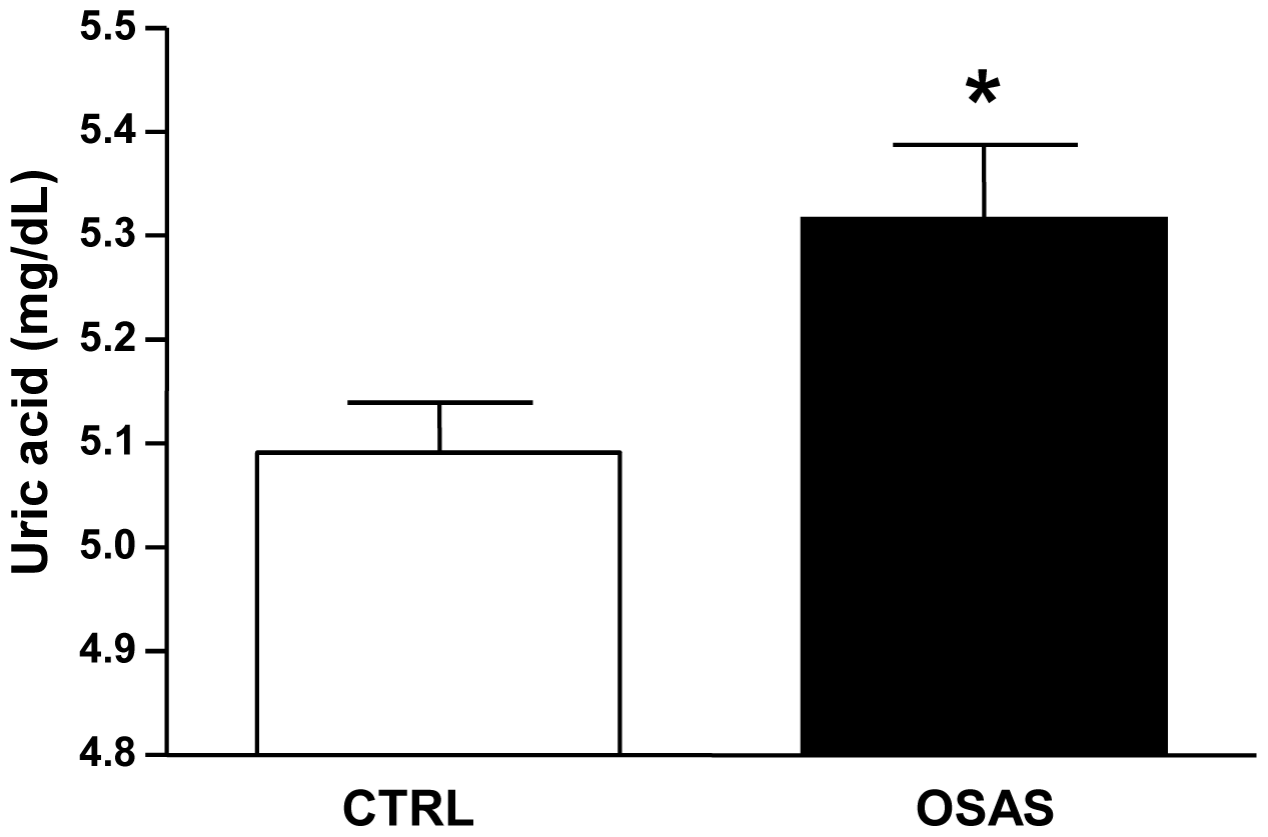
Adjusted mean±SEM of uric acid levels (mg/dL) covariated by body mass index (BMI)-categorical, age-categorical, gender, race-ethnicity, social class and other cardiovascular risk factors (SBP, cholesterol, LDL, HDL, triglycerides and glicemia) in control (CTRL) and obstructive sleep apnea syndrome (OSAS) groups. *p<0.05 compared to CTRL group.

Table 1 illustrates the most important clinical and demographic data in CTRL and OSAS groups (not controlled for confounder variables). Figure 1 shows that the ANOVA revealed group (F_1,1017_ = 47.06; p<0.0001) and gender (F_1,1017_ = 356.83; p<0.0001) effects, showing that uric acid levels were higher in the OSAS group compared to the control (CTRL) group independent from gender. Also, the levels of uric acid were always higher in men than in women. However, after controlling for BMI and age, the group effect was no longer significant. As the groups were not BMI- and age-matched, we determined the cut-offs for these covariates, i.e., the values from which they statistically affected the analysis as a confounding variable. Thus, these values were used to stratify the population sample into binary groups and reduce or eliminate the influence of BMI and age in the analysis to better describe the results. Thus, using ANCOVA, the values of the covariates appearing in the model were 26.8 kg/m^2^ for BMI and 43 years old for age. Next, we categorized these variables in 2 bands (15.1–26.8 kg/m^2^ and 26.9–55.0 for BMI-categorical; 20–43 years old and 44–80 years old for age-categorical). Then, following the same rationale, we proceeded to perform another ANCOVA test controlling for BMI-categorical, age-categorical, gender, ethnicity, social class and other cardiovascular risk factors (SBP, cholesterol, LDL, HDL, triglycerides and glucose). The results still revealed a significant group effect (F_1,956_ = 3.65; p<0.05), confirming that the OSAS group presented higher levels of uric acid compared to the CTRL group even after control for all confounding factors (Figure 2).

**Table 1:** Clinical presentation and demographic parameters in the epidemiological sample of Sao Paulo according to OSAS and CTRL groups (N = 1021).

	CTRL	SAOS	*p*
**Clinical presentation**			
Age (years)	38.4±0.5	50.8±0.7*	<0.0001
BMI (kg/m^2^)	25.4±0.2	29.7±0.3*	<0.0001
SBP (mmHg)	119.8±1.0	135.5±1.5*	<0.0001
DBP (mmHg)	76.9±0.6	84.0±0.9*	<0.0001
Apnea-hypopnea index	1.9±0.1	20.5±0.9*	<0.0001
Uric acid (mg/dL)	4.9±0.1	5.7±0.1*	<0.0001
**Gender (%)**			
Men	25.9	18.8*	<0.0001
Women	40.9	14.4	
**Ethinicity (%)**			
Caucasian	36.2	21.5*	
Afro-Brazilian	9.4	4.2	
Mulatto	9.8	2.4	<0.01
Asian	2.1	0.8	
Native	2.2	0.9	
Unknown/other	7.0	3.4	
**Socioeconomic status (%)**			
High-income	16.6	11.8*	
Middle-income	43.1	18.8	<0.001
Low-income	7.1	2.6	

SBP = systolic blood pressure; DBP = diastolic blood pressure; BMI = body mass index.

* mean or frequency significantly increased when compared to respective CTRL group.

**Figure 3:**
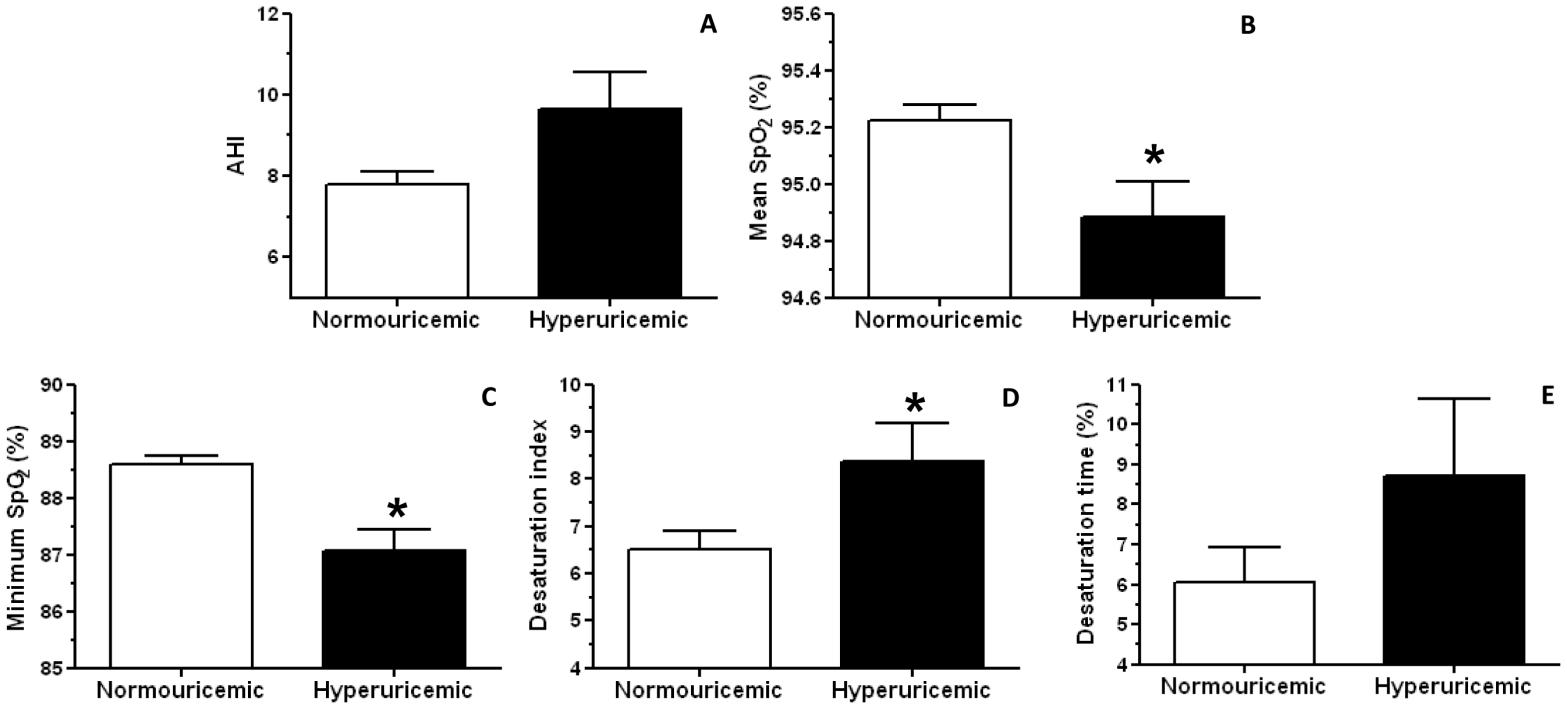
Adjusted mean±SEM of apnea-hypopnea Index (AHI, n = 1020, A), mean oxyhemoglobin saturation (SpO_2_, n = 1020, B), minimum SpO_2_ (n = 1020, C), desaturation index (n = 843, D) and desaturation time (n = 996, E) covariated by gender, body mass index (BMI)-categorical and age-categorical in normouricemic and hyperuricemic groups. Data are shown as Mean ± SEM. *p<0.05 compared to normouricemic group.

To further evaluate the relationship between uric acid levels and OSAS, we first categorized the uric acid variable into high or low levels. In this sense, hyperuricemia was defined by the concentration ≥95th percentile of the normal distribution curve by gender. The 95% uric acid values were 7.0 mg/dL in men and 5.7 mg/dL in women. Overall, the prevalence of hyperuricemia was 16.8%, higher in men (9.8%) than in women (7.0%, χ^2^ = 15.20, df = 1, p<0.0001). Figure 3 shows the effects of uricemia, controlled for BMI-categorical, age-categorical, and gender on 5 variables strongly associated with OSAS: AHI (A), mean SpO_2_ (B), minimum SpO_2_ (C), desaturation index (D) and desaturation time (E). Despite the trend for increased AHI in the hyperuricemic compared to normouricemic group, no significant differences were observed (F_1,1015_ = 3.45, p = 0.06) (Figure 3A). Importantly, ANCOVA analysis revealed an effect of uricemia on the mean SpO_2_ (F_1,1015_ = 6.14, p<0.05), demonstrated by a reduction in the hyperuricemic compared to the normouricemic group (Figure 3B). Similarly, a significant uricemia effect was found on the minimum SpO_2_ (F_1,1015_ = 13.32, p<0.0001), showing a decrease in hyperuricemic compared to normouricemic group (Figure 3C). Regarding the desaturation index, ANCOVA analysis also demonstrated an uricemia effect (F_1,838_ = 4.13, p<0.05) with increased desaturation index in the hyperuricemic group when compared to the normouricemic (Figure 3D). No differences were found in the desaturation time (F_1,991_ = 1.54, p>0.05) (Figure 3E).

**Figure 4:**
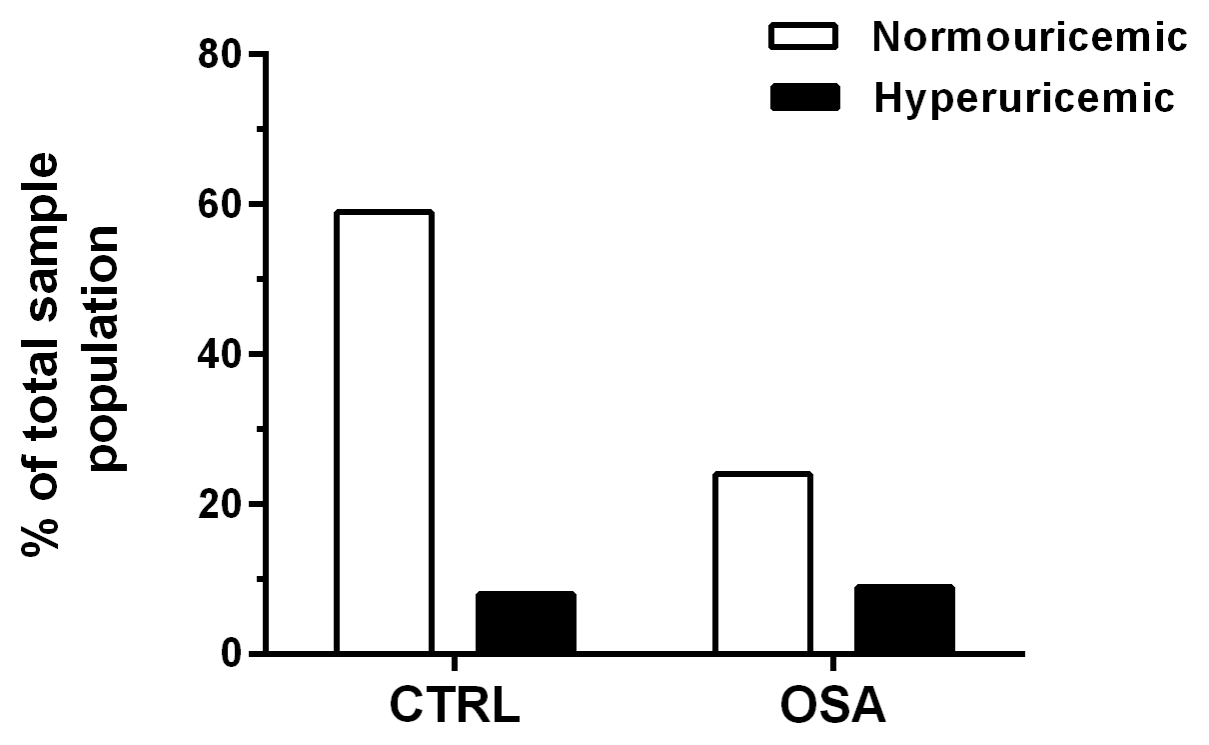
Frequency of normouricemia (n = 849) and hyperuricemia (n = 172) in control (CTRL, n = 682) and obstructive sleep apnea syndrome (OSAS, n = 339) volunteers. Significant association between OSAS and hyperuricemia.

In addition, we found a significant association between hyperuricemia and the presence of OSAS (χ^2^ = 40.6, df = 1, p<0.0001, N = 1021). Figure 4 shows that 54% of hyperuricemic volunteers also had OSAS compared to 29% of normouricemic volunteers. To establish the possible predictors of uric acid levels that could be contributing to the association of hyperuricemia and OSAS, we calculated a correlation matrix between uric acid and all sleep, physical and circulating parameters collected (data not shown). Table 2 shows the correlation coefficients of uric acid levels with the most relevant risk factors after the analysis, which were: age, AHI, arousal index, BMI, total cholesterol and fractions, creatinine, desaturation index and time, glucose, minimum SpO_2_, SBP, Na^+^ and triglycerides. Overall, we observed a slight positive correlation of uric acid levels with age, arousal index, total cholesterol, desaturation index, glucose, LDL and Na^+^ in the total population. Moreover, a mild positive correlation was observed between uric acid and AHI, BMI, SBP and triglycerides. On the other hand, a mild negative correlation was found between uric acid and HDL and minimum SpO_2_. A moderate positive correlation was only observed between uric acid levels and creatinine in the total population sample (R = 0.49, p<0.0001). Next, a multiple linear regression model was performed to identify the possible predictors of uric acid levels in men, women and the total population sample. Importantly, Table 3 shows that in men triglycerides, creatinine, SBP, K^+^ and minimum SpO_2_ entered into the model explaining 22% of uric acid variability (R = 0.47, R^2^ = 0.22, F_5,437_ = 24.63, p<0.0001, Table 3). In women, the BMI>26.8 kg/m^2^, creatinine, minimum SpO_2_, triglycerides and SBP entered into the model explaining 27% of uric acid variability (R = 0.52, R^2^ = 0.27, F_5,544_ = 39.32, p<0.0001, Table 4). Considering the total population, the significant predictors of uric acid levels were gender (men), creatinine, triglycerides, BMI>26.8 kg/m^2^, SBP, minimum SpO_2_, and K^+^ level (R = 0.68, R^2^ = 0.46, F_7,982_ = 120.63, p<0.0001, Table 5).

**Table 2:** Pearson’s correlation coefficients between serum uric acid and risk factors distributed by gender.

	Men			Women			Total population		
	*R*	*p*	*N*	*R*	*P*	*N*	*R*	*P*	*N*
**Age**	0.17	<0.0001	456	0.27	<0.0001	565	0.13	<0.0001	1021
**AHI**	0.16	<0.0001	456	0.22	<0.0001	565	0.25	<0.0001	1021
**Arousal index**	0.11	<0.05	456	0.13	<0.01	565	0.19	<0.0001	1021
**BMI**	0.35	<0.0001	455	0.45	<0.0001	565	0.31	<0.0001	1021
**Cholesterol**	0.21	<0.0001	456	0.18	<0.0001	565	0.14	<0.0001	1021
**Creatinine**	0.23	<0.0001	456	0.26	<0.0001	565	0.49	<0.0001	1021
**Desaturation index**	0.15	<0.0001	416	0.24	<0.0001	428	0.21	<0.0001	844
**Desaturation time**	0.10	<0.05	445	0.14	<0.001	552	0.11	<0.0001	997
**Glucose**	0.14	<0.0001	455	0.16	<0.0001	565	0.15	<0.0001	1020
**HDL**	−0.17	<0.0001	456	−0.16	<0.0001	565	−0.34	<0.0001	1021
**LDL**	0.10	<0.05	444	0.16	<0.0001	563	0.11	<0.0001	1007
**Minimum SpO_2_**	−0.20	<0.0001	456	−0.33	<0.0001	565	−0.28	<0.0001	1021
**SBP**	0.27	<0.0001	441	0.29	<0.0001	551	0.28	<0.0001	992
**Sodium**	0.07	>0.05	454	0.11	<0.01	559	0.11	<0.001	1013
**Triglycerides**	0.33	<0.0001	456	0.29	<0.0001	565	0.37	<0.0001	1021

**Table 3:** Multiple linear regression for uric acid levels predictors in men (n = 437). SBP = systolic blood pressure; SpO_2_ = oxyhemoglobin saturation.

	*β*	*t*	*p*
**Constant**		8.08	<0.0001
**Triglycerides**	0.28	6.55	<0.0001
**Creatinine**	0.20	4.49	<0.0001
**SBP**	0.16	3.44	<0.001
**Potassium**	− 0.13	− 2.93	<0.01
**Minimum SpO_2_**	− 0.13	− 2.84	<0.01

**Table 4:** Multiple linear regression for uric acid levels predictors in women (n = 550).

	*β*	*t*	*p*
**Constant**		−10.19	<0.0001
**BMI>26.8 kg/m^2^**	0.25	5.79	<0.0001
**Creatinine**	0.20	5.38	<0.0001
**Minimum SpO_2_**	− 0.15	− 3.56	<0.0001
**Triglycerides**	0.12	3.11	<0.01
**SBP**	0.11	2.71	<0.01

SBP = systolic blood pressure; SpO_2_ = oxyhemoglobin saturation; BMI = body mass index.

**Table 5:** Multiple linear regression for uric acid levels predictors the total sample population (n = 992).

	*β*	*t*	*p*
**Constant**		10.10	<0.0001
**Gender (men)**	0.36	12.23	<0.0001
**Creatinine**	0.21	7.04	<0.0001
**Triglycerides**	0.18	7.18	<0.0001
**BMI>26.8 kg/m^2^**	0.13	5.03	<0.0001
**SBP**	0.11	4.30	<0.0001
**Minimum SpO_2_**	− 0.10	− 3.82	<0.0001
**Potassium**	− 0.06	− 2.61	<0.01

SBP = systolic blood pressure; SpO_2_ = oxyhemoglobin saturation; BMI = body mass index.

**Figure 5:**
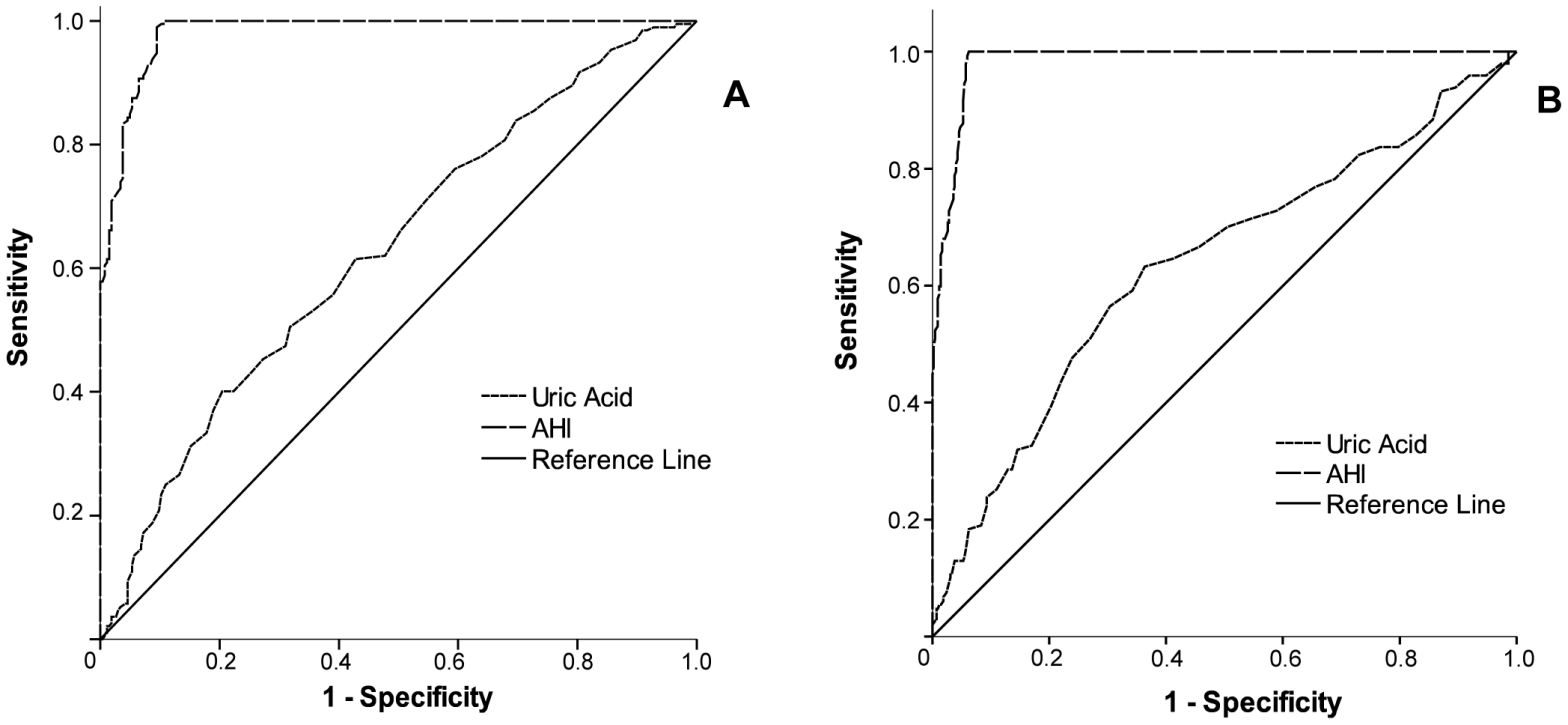
Receiver-operating characteristics (ROC) curve analysis comparing serum uric acid levels and apnea-hypopnea index (AHI) as classifiers for obstructive sleep apnea syndrome (OSAS) diagnostic.

Finally, to verify whether uric acid levels could be, in turn, a risk factor for OSAS in our sample population, a binary logistic regression analysis was performed. Table 6 depicts the model with the most favorable Hosmer-Lemeshow goodness-of-fit (χ^2^ = 4.48, df = 8, p>0.05) and the corresponding odds ratio (OR) of each independent predictor of OSAS (−2LL = 961.50; χ^2^ = 274.24, df = 9, p<0.0001; Cox-Snell R = 0.25; Nagelkerke R = 0.34): age>43 years, BMI>26.8 kg/m^2^, gender (men) and uric acid levels. Importantly, the results show that an increase in 1 mg/dL in uric acid level was associated with 16% increased risk of OSAS (95% C.I. = 1.01–1.33). The other variables, SBP, cholesterol, HDL, LDL and triglycerides, did not enter the model. Figure 5 and Table 7 show the areas under the ROC curves (AURC) and the optimal cut-off points (according to the highest sensitivity and specificity) of uric acid levels associated with OSAS diagnosis. The best cut-offs were 5.95 mg/dL for men and 4.45 mg/dL for women, which were in the 50^th^ percentile of the uric acid distribution curve and corresponded respectively to a positive predictive value (PPV) and negative positive value (NPV) of 61.0% and 55.7% in men and 37.8% and 82.1% in women.

**Table 6:** Binary logistic regression model for calculation of the odds ratio (OR) related to the obstructive sleep apnea syndrome (OSAS) condition.

	*OR*	*p*	*95% C.I. for OR*	
			*Lower*	*Upper*
**Constant**	0.001	<0.0001	–	–
**Uric acid**	1.16	<0.05	1.01	1.33
**Age>43 years**	4.52	<0.0001	3.18	6.42
**BMI>26.8 kg/m^2^**	3.21	<0.0001	2.28	4.52
**Gender (men)**	2.58	<0.0001	1.71	3.87
**SBP**	–	>0.05	1.00	1.01
**Cholesterol**	–	>0.05	0.35	1.05
**HDL**	–	>0.05	0.95	2.84
**LDL**	–	>0.05	0.96	2.86
**Triglycerides**	–	>0.05	0.99	1.23

Adjusted model for the confounding factors: age>43 years, body mass index (BMI)>26.8 kg/m^2^, gender (reference for men), systolic blood pressure (SBP), cholesterol, LDL, HDL and triglycerides (N = 977).

**Table 7:** Areas under the ROC curves (AURC), cut-offs, sensitivity and specificity of serum uric acid levels in relation to the ability to identify obstructive sleep apnea syndrome (OSAS).

	Variable	AURC	SEM	*p*	*95% C.I.*	*Cut off*	*Sensitivity*	*Especificity*
**Men**	**Uric acid**	0.63	0.026	<0.0001	0.58–0.68	5.95	0.62	0.57
	**AHI**	0.98	0.005	<0.0001	0.97–0.99			
**Women**	**Uric acid**	0.64	0.028	<0.0001	0.58–0.69	4.45	0.63	0.64
	**AHI**	0.98	0.004	<0.0001	0.98–0.99			

## Discussion

This is the first study showing the influence of OSAS on uric acid levels in an epidemiological sample. Interestingly, the results indicate that individuals diagnosed with OSAS had higher levels of serum uric acid than those without OSAS. Additionally, this effect remained significant after adjustment for confounding factors such as gender, age, BMI, social class, ethnicity, cholesterol, triglycerides, blood pressure and glucose, unlike the data published by García et al. [25]. Moreover, a higher prevalence of hyperuricemia was found in men, which is also the gender with more prevalence of OSAS [1], [2].

We also demonstrated important associations between uric acid levels and common OSAS-related risk factors such as SpO_2_, AHI, arousal index, desaturation, cholesterol and fractions, triglycerides, SBP and BMI in this large sample. Similarly, our data showed that hyperuricemic volunteers, regardless of OSAS diagnosis, presented lower mean and minimum SpO_2_, and higher desaturation index during sleep, explaining the strong association found between hyperuricemia and OSAS. Additionally, uric acid was considered an independent predictor of OSAS, being associated with 16% increased risk of OSAS with each increase of 1 mg/dL in its concentration. Conversely, minimum SpO_2_ entered the multiple linear regression model as an independent predictor of uric acid levels explaining 10% of its variability, along with other factors such as gender (men), creatinine, triglycerides, BMI>26.8 kg/m^2^, SBP and serum K^+^ levels. On the other hand, ROC curve analysis revealed that, in both men and women, uric acid levels cut-off points determined through its highest sensitivity and specificity did not qualify for a good biomarker of OSAS, presenting poor PPV and NPV.

A large body of evidence has identified OSAS as an independent risk factor for cardiovascular morbidity and mortality with multiple associated mechanisms [8]. One of them is the oxidative stress [36], which is also a common pathway linked to uric acid production, a process catalyzed by xanthine oxidase [37]. During the production of uric acid, ROS are generated as byproduct and have an important role in the increased vascular oxidative stress. Importantly, xanthine oxidase mediates intermittent hypoxia-induced vascular dysfunction and administration of allopurinol can prevent it by increasing intermediates, hypoxanthine and xanthine, and decreasing the final product uric acid [38], [39]. In fact, oxidative stress is reflected by uric acid levels, and its concentration decreases after treatment of sleep disordered breathing according to improvement of respiratory disturbance index and oxygen desaturation index [40]. Elevated serum uric acid levels are also associated with increased risk for cardiovascular mortality [21], [41], [42]. In fact, most of the cardiovascular risk factors potential overlaps with serum uric acid levels concentrations [43], [44], [45]. In this sense, a positive association of hyperuricemia with obesity, impaired glucose tolerance, hypertension, history of heart disease and mortality was observed in a large Finland cohort population (aged 40–69) [43]. Moreover, some studies have reported that elevated levels of uric acid are associated not only with the presence of cardiovascular disease but also with poor prognosis of stable coronary artery disease [46], acute myocardial infarction [47], [48], [49], heart failure [50], stroke [51], and metabolic syndrome [52].

Corroborating Jubber and colleagues [53], we also found an association between BMI and elevated blood concentrations of uric acid. Both hyperuricemia and excessive accumulation of body fat are known to be associated with greater cardiovascular risk and oxidative stress [25], [26], [54], suggesting that uric acid may have an important role in mediating OSAS in obese patients. Similarly, Verhulst et al. [54] have demonstrated a relationship between the severity of sleep apnea and increased levels of serum uric acid in overweight children and adolescents, independent of abdominal adiposity. In fact, obese individuals present a higher incidence of respiratory diseases, including OSAS, which contributes to increased morbidity and mortality among this population [55], [56].

Confirming our hypothesis, the linear regression model presented in the current study showed important participation of the minimum SpO_2_ in the uric acid levels even after control for confounders such as BMI. Moreover, triglycerides and hypertension also participated as important predictors of the uric acid levels in agreement with some previews studies [57], [58]. Recently, Desai et al. [59] showed that a significant linear increase in uric acid levels occurs with increasing metabolic risk factors in middle-aged Brazilian men. Patients who had a high triglyceride/HDL ratio had significantly higher serum uric acid levels than did those who had a normal ratio. Taken together, these results reflect a close relationship between uric acid levels and metabolic syndrome. However, it is important to stress that the tissue hypoxia caused by OSAS is determined by a complex balance between the supply of arterial oxygen and tissue oxygen demand. Thus, arterial oxygen supply depends on other factors besides oxyhemoglobin saturation, including hemoglobin concentration, the oxygen-hemoglobin dissociation curve and cardiac output.

Similar to our data, some studies found increased uric acid levels in OSAS patients compared to controls in addition to higher levels of blood pressure, glucose, triglycerides, cholesterol and LDL [60], [61]. Drager et al. [61] showed that OSAS was also independently associated with increased uric acid, but with a OR of 4.19 (1.70–10.35), much higher than we found in the current study, likely due to the different populations studied (all patients selected by Drager et al. presented metabolic syndrome). Moreover, similar to our findings, Kaditis et al. [62] found that AHI and SpO_2_ were related to uric acid excretion in Greek (but not US) children even after adjustments for age, gender, and BMI Z-score. Another study also found a significant correlation between uric acid levels and some sleep,parameters (number of respiratory events, number of desaturations, or the cumulative percentage of time with oxygen saturation less than 90%) [25]. Additionally, they showed that those patients with severe OSAS (AHI≥30) had higher uric acid levels than those with mild or no OSAS. However, this difference was not persistent after control for the confounding factors BMI, cholesterol and triglyceride levels [25]. Likewise, Hira et al. [63] found a significant linear relationships of AHI with uric acid while Steiropoulos and colleagues [64] showed that OSAS patients with good compliance to CPAP presented a decrease in uric acid levels after CPAP treatment when compared to those with bad compliance.

Uric acid has been studied in several cardiorespiratory processes that produce hypoxia since this condition leads to increased catabolism of purines. In this sense, uric acid has shown useful as a prognostic marker of heart failure [65], pulmonary thromboembolism [66], and primary pulmonary hypertension [19]. A recent study in chronic obstructive pulmonary disease patients using the ratio between uric acid and creatinine has been shown to be more useful than the use of serum uric acid levels alone [67]. However, in our study we did not find the same result, since no significant differences were found using the uric acid/creatinine ratio (data not shown). Moreover, two studies using this method in OSAS patients presented different results. Braghiroli et al. [68] concluded that uric acid/creatinine ratio could be a promising index of significant nocturnal tissue hypoxia, since their OSAS patients had a marked reduction in the uric acid/creatinine ratio after treatment with CPAP. On the other hand, McKeon et al. [69], studying hypoxemic patients with OSAS before and after CPAP treatment, did not find significant differences in the uric acid/creatinine ratio. In addition, another study showed that the index of uric acid/creatinine from OSAS patients did not parallel the severity of the AHI or arterial oxygen desaturation, but it was significantly linked to the plasma level of adenosine, which is another marker of tissue hypoxia [12].

Our data indicate that increased uric acid may be an important risk factor for the severity of OSAS, possibly by favoring oxidative stress and endothelial dysfunction. Conversely, minimum SpO_2_ decrease, resultant from OSAS-hypoxia/reoxygenation cycles, is an important predictor of uric acid levels and may be responsible for the association found between hyperuricemia and OSAS in our epidemiological sample of Sao Paulo. Nevertheless, the best cut-offs for uric acid level as a classifier of OSAS presented around 60% sensibility and 60% specificity, indicating that it fails to be a good biomarker by itself. Probably, a combination of OSAS-related biological analytes rather than only one would be more applicable as a potential biomarker.

Finally, the current study suggests that uric acid measurement may be important in the management of OSAS and its comorbidities, mainly where people do not have access to health-care facilities and sleep laboratories. Since uric acid overproduction is accompanied by an enhanced synthesis of ROS, the current study may contribute to the mechanisms linking sleep-disordered breathing and cardiovascular risk. However, long-term epidemiological studies to verify the consequences of decreasing uric acid concentration in OSAS patients and also to evaluate uric acid levels changes after CPAP treatment are needed.
